# Supplementary material for: Cell type signatures in cell-free DNA fragmentation profiles reveal disease biology
Source: Nat Commun. 2024 Mar 12;15:2220. doi: 10.1038/s41467-024-46435-0 (PMC10933257; doi:10.1038/s41467-024-46435-0)

Supplementary Information

Cell type signatures in cell free DNA fragmentation profiles reveal disease biology

Kate E Stanley[1,2], Tatjana Jatsenko[1], Stefania Tuveri[1], Dhanya Sudhakaran[1], Lore Lannoo[3], Kristel Van Calsteren[3], Marie de Borre[4], Ilse Van Parijs[5], Leen Van Coillie[5], Kris Van Den Bogaert[5], Rodrigo De Almeida Toledo[6], Liesbeth Lenaerts[7], Sabine Tejpar[8], Kevin Punie[9], Laura Y. Rengifo[10], Peter Vandenberghe[10,11], Bernard Thienpont[4], Joris Robert Vermeesch*[1]

[1]Department of Human Genetics, Laboratory for Cytogenetics and Genome Research, KU Leuven, Leuven, Belgium.
[2]Department of Biosciences and Nutrition, Karolinska Institute, Sweden
[3]Department of Gynecology and Obstetrics, University Hospitals Leuven, Leuven, Belgium.
[4]Department of Human Genetics, Laboratory for Functional Epigenetics, KU Leuven, Leuven, Belgium.
[5]Center for Human Genetics, University Hospitals Leuven, Leuven, Belgium.
[6]Vall d'Hebron Institute of Oncology, Barcelona (VHIO), Spain
[7]Department of Oncology, Gynecological Oncology, KU Leuven, Leuven, Belgium.
[8]Department of Oncology, Molecular Digestive Oncology, KU Leuven, Leuven, Belgium.
[9]Multidisciplinary Breast Centre, Leuven Cancer Institute, University Hospitals Leuven, Leuven, Belgium.
[10]Department of Human Genetics, Laboratory of Genetics of Malignant Diseases, KU Leuven, Leuven, Belgium.
[11]Department of Hematology, University Hospitals Leuven, Leuven, Belgium.

*Correspondence:
Prof. Joris Robert Vermeesch
Center for Human Genetics, University Hospitals Leuven
Herestraat 49, box 602, Leuven 3000, Belgium
Telephone: +32 16 34 5941
Email:joris.vermeesch@kuleuven.be

Supplementary Table 1: Case-control sex characteristics for cancer cohorts. Two-way contingency tables for the number of female and male individuals in the case and control groups presented in the study. A Fisher exact test was used to test for an association between sex and case-control status for each cohort and the exact p-value is reported. A p-value cutoff of 0.05 was used to determine significance.

	Female	Male
**Colorectal Cancer (10X)**	7	9
**Controls (10X)**	88	51

The Fisher exact p-value is 0.1751. The result is *not* significant at $p < .05$.

	Female	Male
**Breast Cancer (10X)**	52	0
**Controls (10X)**	88	0

The Fisher exact p-value is 1. The result is *not* significant at $p < .05$.

	Female	Male
**Multiple Myeloma (<0.3X)**	12	12
**Controls (<0.3X)**	34	56

The Fisher exact p-value is 0.3502. The result is *not* significant at $p < .05$.

Supplementary Figure 1: Nucleosome peak calls in cfDNA data sequenced at different depths are concordant with an *in silico* callset. Genomic sequences have different affinities for histones and contribute to the assemply and positioning of nucleosomes *in vivo*. Kaplan *et al* (2009) published an *in silico* model that predicts nucleosome positions based on genomic sequence. Their per-base probabilities for nucleosome occupancy were calculated using the hg19 assembly. Therefore, we applied the *in silico* model from Kaplan *et al* (2009) on 20k gene body coordinates from hg38 (**A)** P occupied for the Kaplan *in silico* model for FCN1 are plotted to illustrate oscillatory patterns. The oscillatory patterns mirror the patterns that we observed in the sequencing coverage and window protection scores (WPS) for our cfDNA data (**B)** We applied the same heuristic peak calling algorithm on the P occupied values as we did on the WPS. For 230 healthy individuals with cfDNA sequencing data, the distance to the nearest peak in the *in silico* callset was distributed around zero for all sequencing depths.

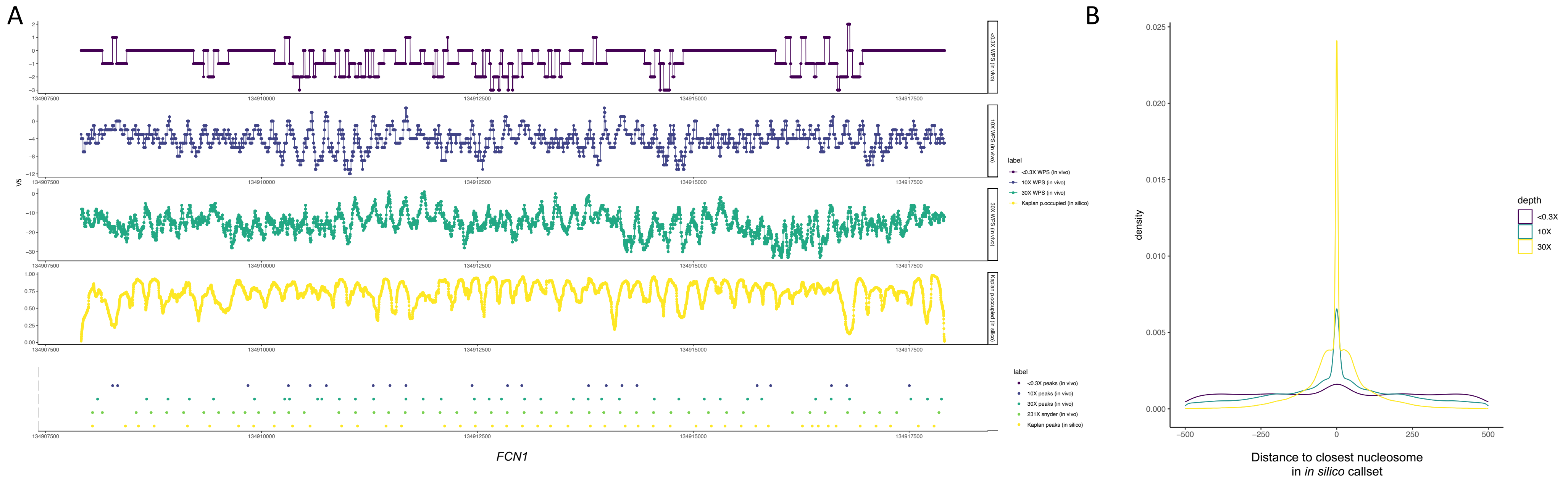

Supplementary Figure 2: Local nucleosome dynamics are recovered from cfDNA data depending on the sequencing depth. We looked at chromatin compartments associated with different functional annotations. To do this we downloaded chromatin state learning maps (ChromHMM v1.10) in primary monocytes, neutrophils, B cells, T cells, and natural killer cells from peripheral blood, as well as maps from eight additional primary tissue types. The ChromHMM core-15 state model uses interactions between 5 different histone marks to predict 15 distinct chromatin states, including quiescence (67.8% genome), transcription (15.2% genome), and transcriptional start sites (TSS) (0.7% genome). Inter-nucleosome distances in transcribed regions compared to quiescent regions in monocytes are shown across **(A)** all samples and **(B)** samples grouped by sequencing depth. **(C)** Internucleosomal distances are shown at specific regulatory sites (i.e. active and bivalent TSS) in samples sequenced at <0.3-fold, 10-fold, and 35-fold coverage grouped by cell type. An enrichment of wider inter-peak distances was observed around TSS that are active in certain blood cells (i.e. monocytes, neutrophils, T cells) compared to TSS active in other primary tissues. This altered distribution was not observed at bivalent TSS that are not bound by transcription factors. The smoothed density curves are red for blood cell types with an enrichment of wider inter-nucleosome distances and grey for the rest of the tissues.

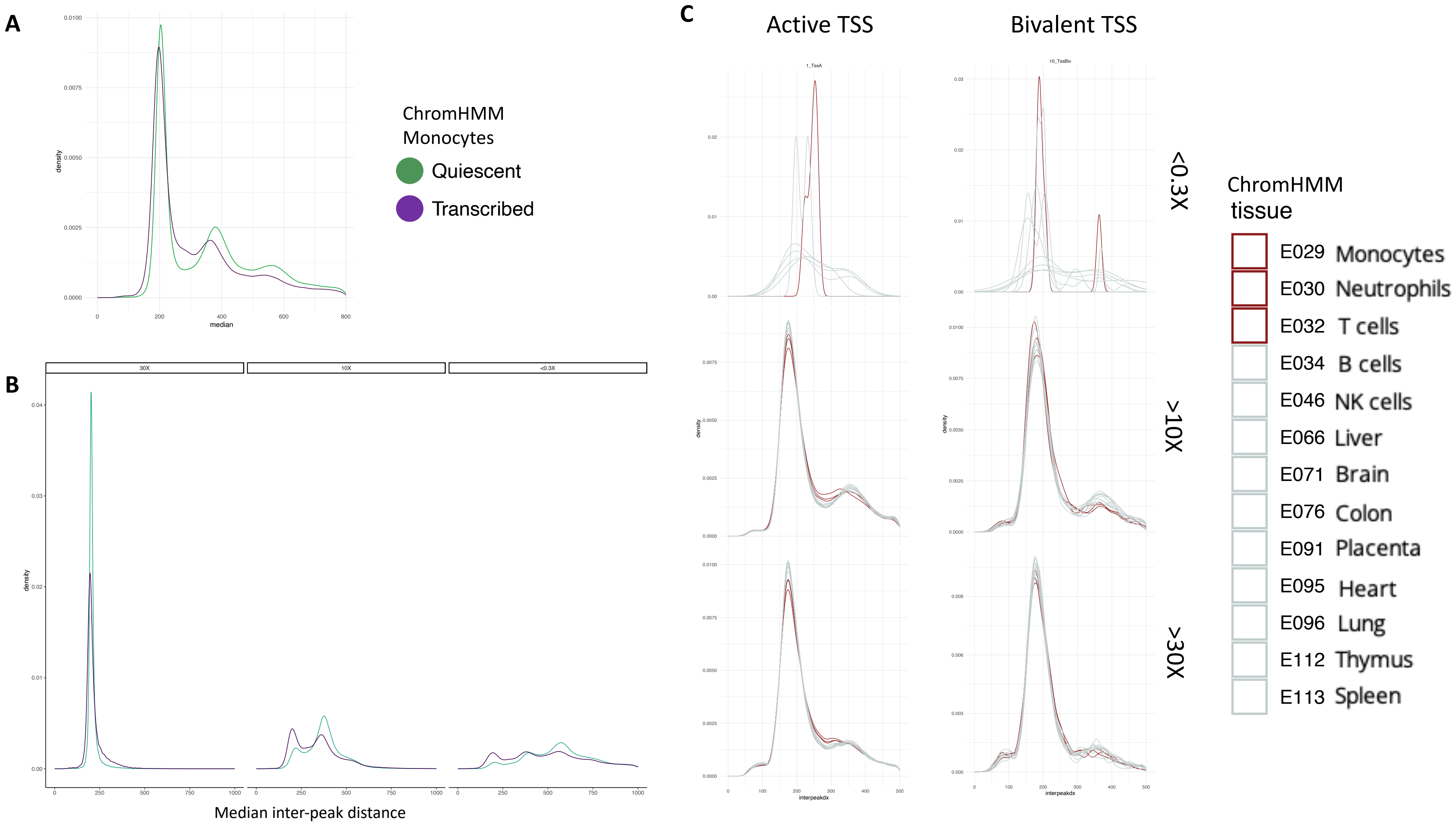

Supplementary Figure 3: Pearson correlation between cell type ranks across down sampling levels for healthy non-pregnant control "GC01". The correlation of 456 cell type (Tabula Sapiens) ranks are shown for each down sampling pair. The y-axis indicates the % of reads down sampled for each level. Pearson correlation values and p-values are provided for each correlation. The down sampling was done on a 35-fold coverage sample, therefore 40% of reads corresponds to 14-fold coverage, 0.5% of reads corresponds to 0.1-fold coverage and 0.1% of reads corresponds to 0.04-fold coverage.

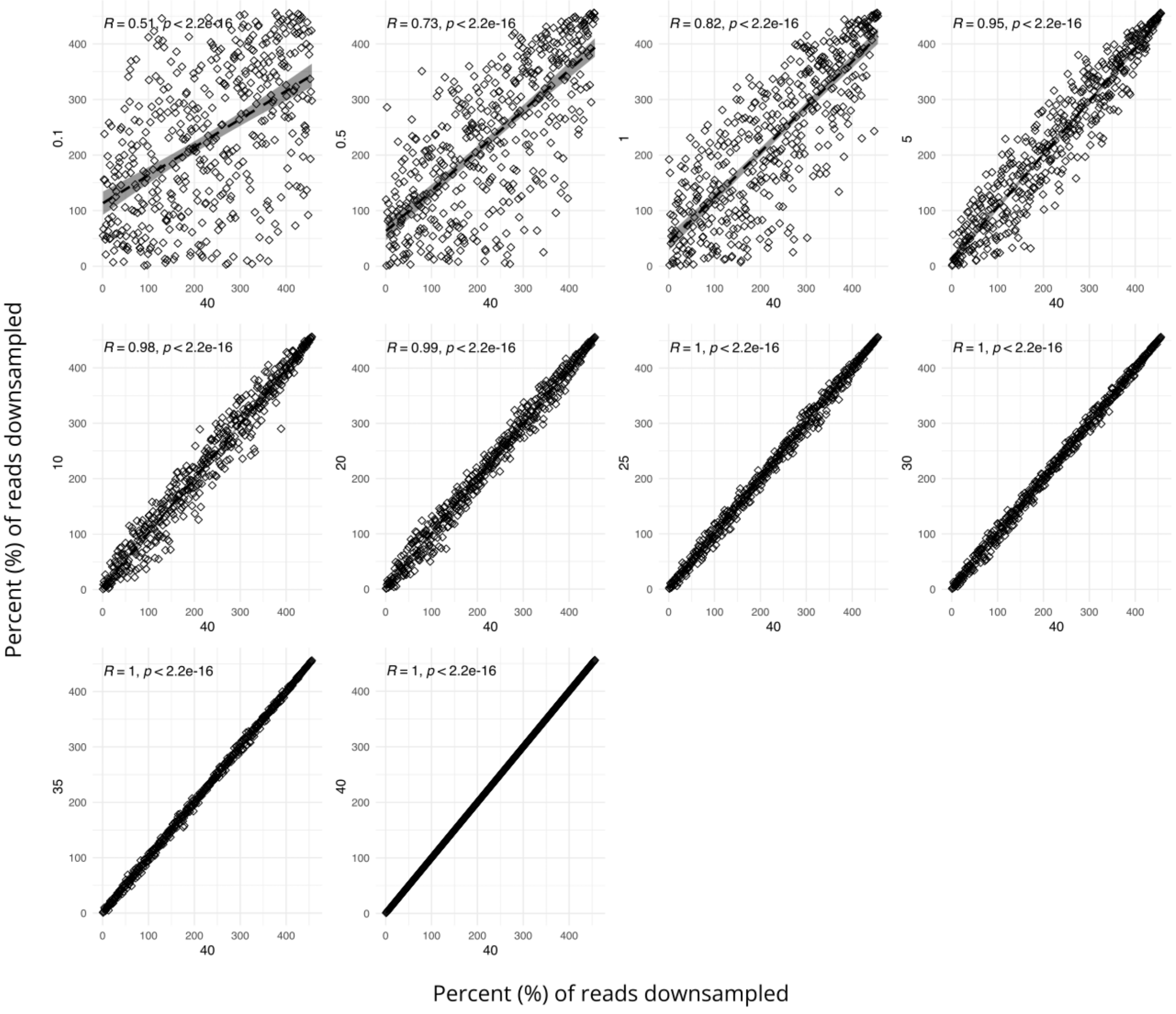

Supplementary Figure 4: Pairwise correlation between cell type ranks across down sampling levels for healthy pregnant control "GC02". The correlation of 487 cell type (Tabula Sapiens + Vento-Tormo) ranks are shown for each down sampling pair. The y-axis indicates the % of reads down sampled for each level. Pearson correlation values and p-values are provided for each correlation. The down sampling was done on a 35-fold coverage sample, therefore 40% of reads corresponds to 14-fold coverage, 0.5% of reads corresponds to 0.1-fold coverage and 0.1% of reads corresponds to 0.04-fold coverage.

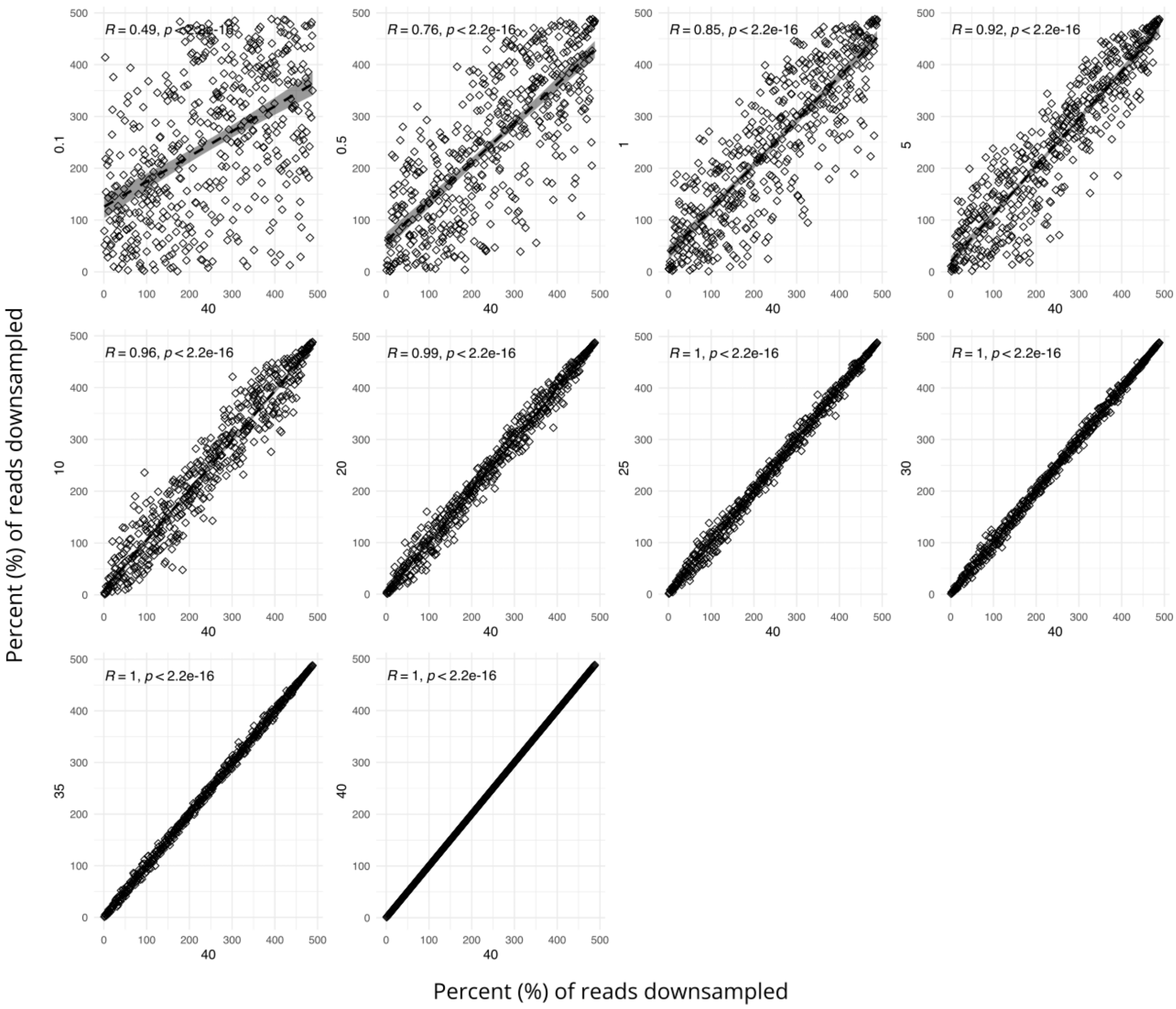

Supplementary Figure 5: Immune cell types shared across tissues differ in their contribution to cfDNA populations. Ranked relative contribution to cfDNA of immune cells stratified by tissue-residency in 230 healthy individuals. This is possible because distinct transcriptional profiles are acquired by certain immune cells in to confer tissue-specific functions. Certain immune cells are only present in a single tissue.

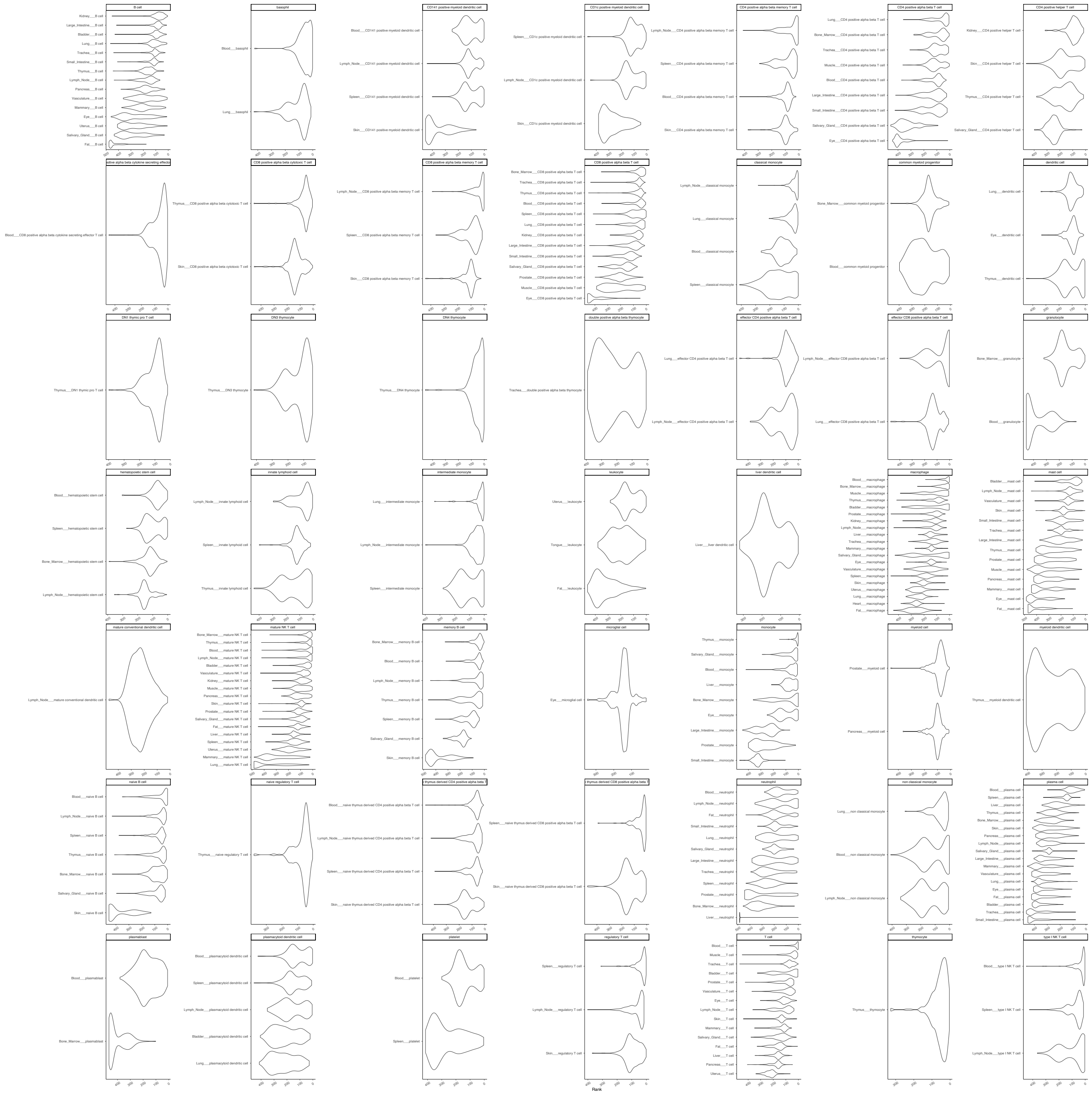

Supplementary Figure 6: Distribution of cell type ranks per tissue in healthy individuals. Rank contribution to cfDNA of all cell types in the reference set (n=456) in 230 healthy individuals. Cell types are grouped by tissue and colored by compartment (i.e. immune, endothelial, epithelial, stromal) as annotated by the original publication.

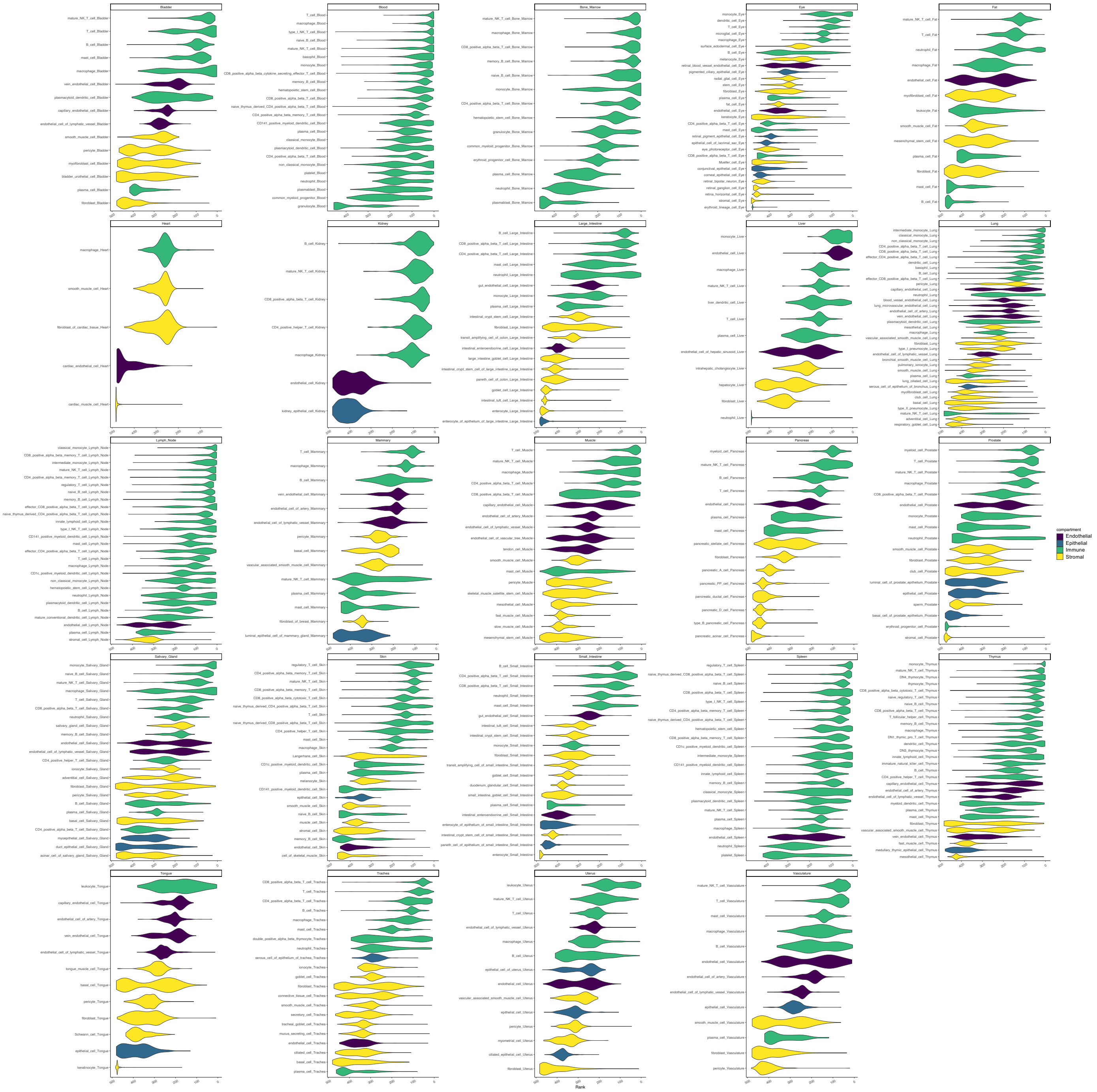

Supplementary Figure 7: Unsupervised clustering and visualization of cell type features in cases and controls. Principal component analysis (PCA) was used for dimension reduction and PCs with eigenvalue > 1 (Kaiser's criterion) were extracted for distance matrix construction using the Euclidean distance, followed by Walktrap community detection to define clusters with fixed parameters (the nearest number of nodes was 3 with a walk step of 2). To visualize the dataset in lower dimensions, t-distributed stochastic neighbor embedding (tSNE) was used. Clusters defined from the community detection were used for tSNE annotation.

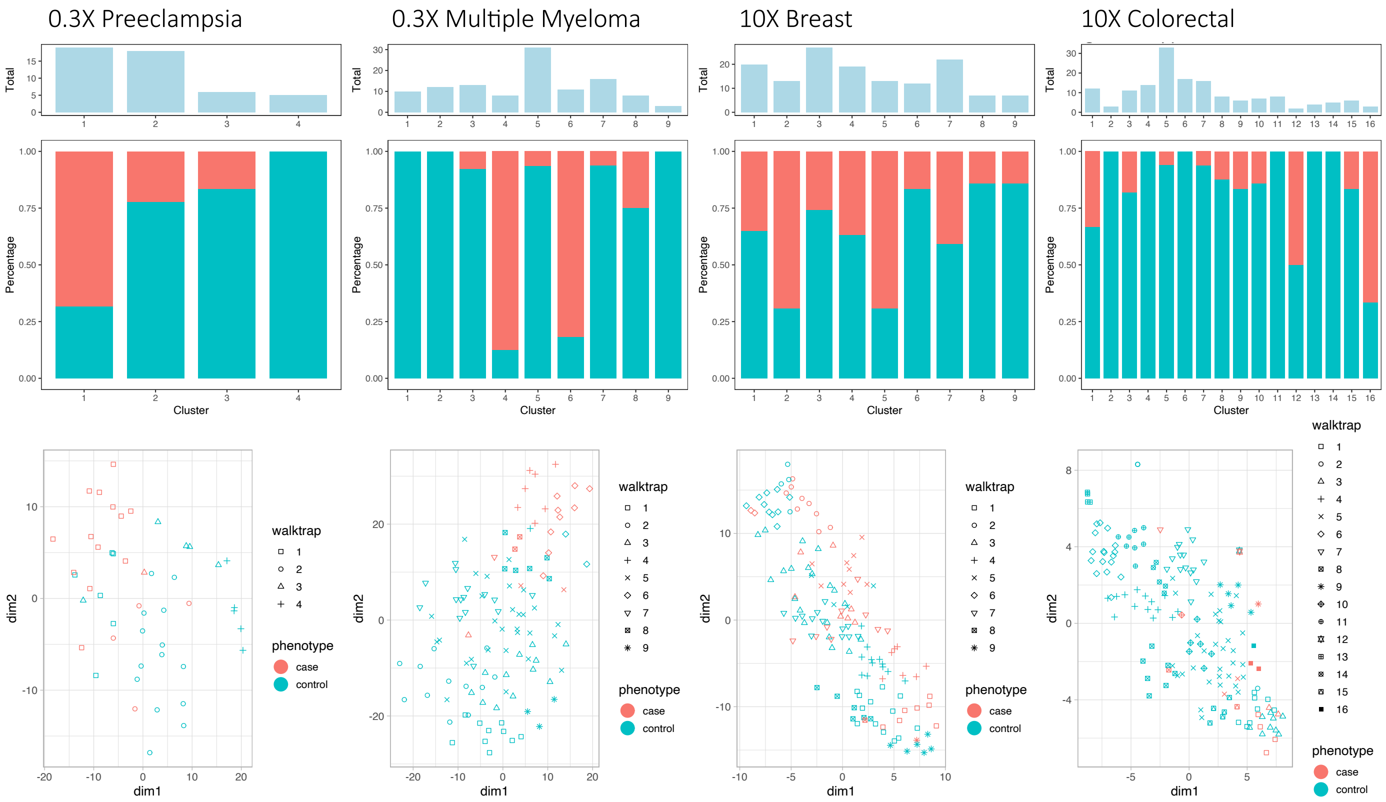

Supplementary Figure 8: Disease prediction using 50 bulk tissue level features. Gene-level fast Fourier transformed (FFT) window protection scores (WPS) were correlated with the consensus transcript levels summarized per gene in 50 tissues from the Human Protein Atlas and GTEx. Tissues were given a rank per sample based on the strength of correlation. A support vector machine with leave-one-out cross validation and default hyperparameters was trained using the resulting tissue rankings for each cancer cohort and preeclampsia cohort (cases + matched controls).

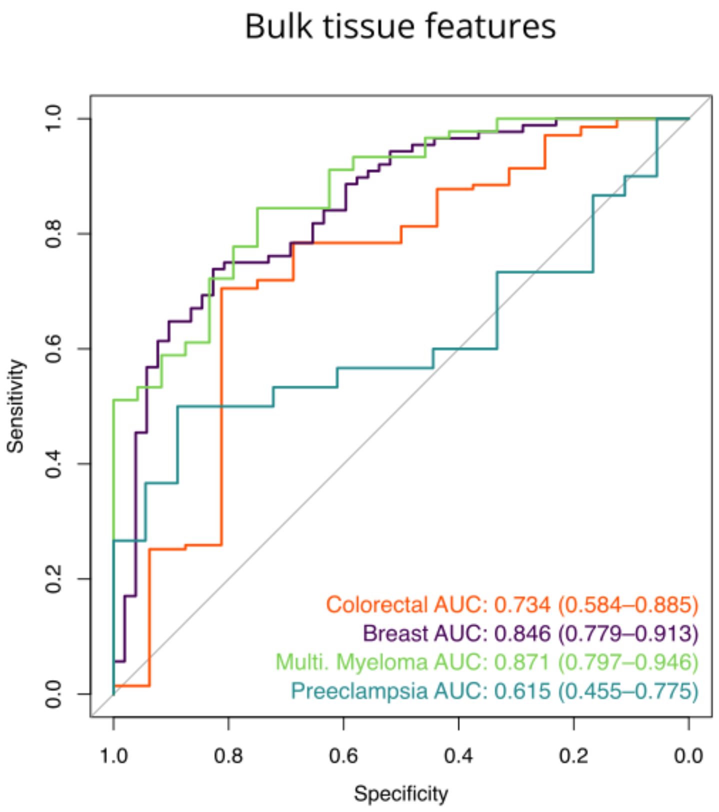

Supplementary Figure 9: Cancer prediction using ichorCNA tumor fractions. IchorCNA tumor fractions were estimated for colorectal cases (n=16), breast cancer cases (n=52), multiple myeloma cases (n=24), and matched high coverage (n=139) and low coverage (n=90) controls using the default panel of normals provided by ichorCNA. Tumor fractions were used as a predictive value in a receiver operating characteristic (ROC) analysis for each cancer type versus matched controls.

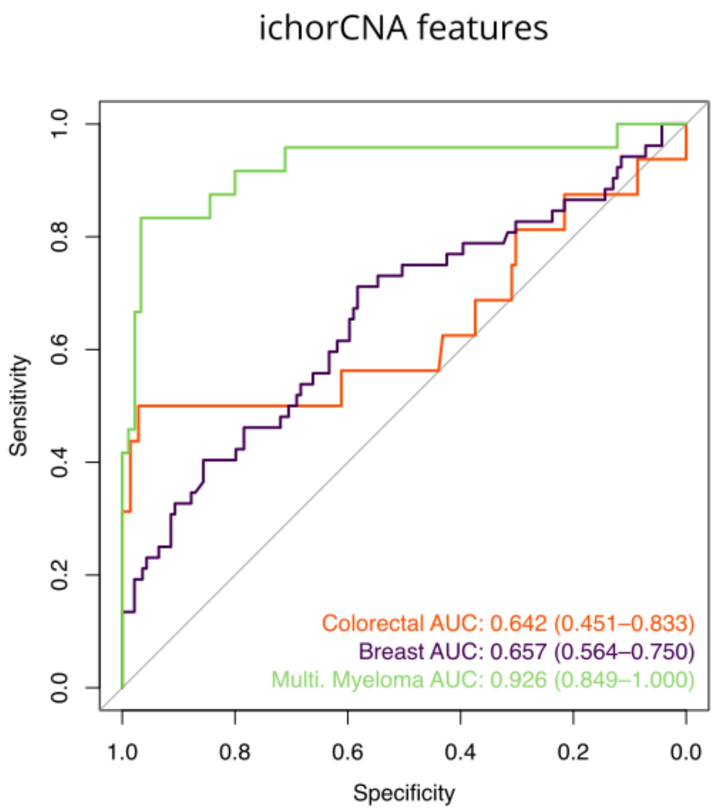

Supplementary Figure 10: Performance of Griffin for cancer prediction. Receiver operating characteristic (ROC) curves for published Griffin model when applied on our <0.3X multiple myeloma cohort, 10X breast cancer cohort, and 10X colorectal cancer cohort. The used the published Griffin model trained on the "LUCAS" cohort available on the Griffin GitHub. The Griffin "LUCAS" model was trained on 1-2X whole-genome sequencing dataset of cfDNA samples from healthy donors (n=158) and cancer patients (n=129).

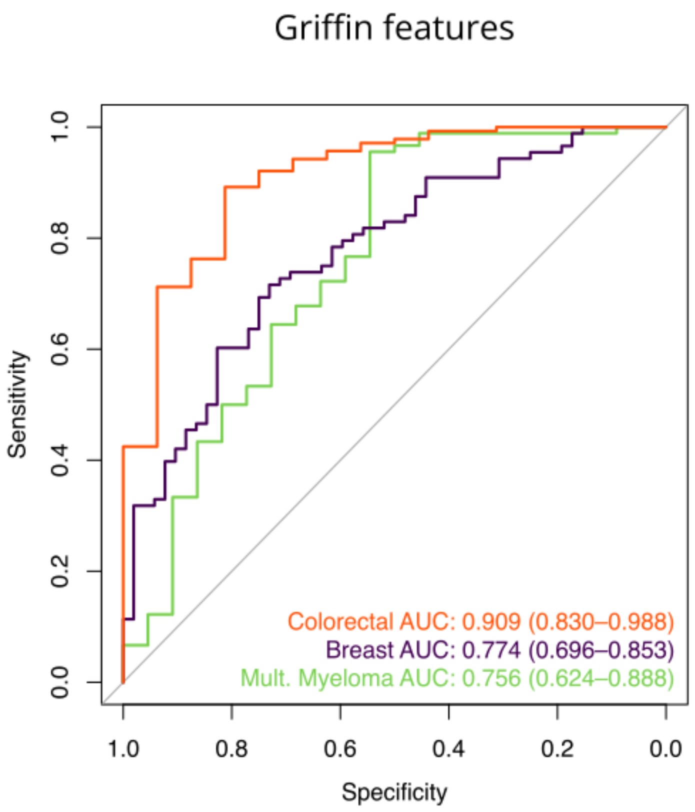

Supplementary Figure 11: Clinical Characteristics of Preeclampsia Cases and Healthy Pregnant Controls. A two-sided Wilcoxon rank sum test between the characteristics of cases and controls for the preeclampsia cohort was used to generate *p*-values. BMI = body mass index. Preg_preeclampsia_diagnosis = pregnant cases sampled at preeclampsia diagnosis. Preg_control_agematched = pregnant control individuals matched to cases by maternal characteristics.

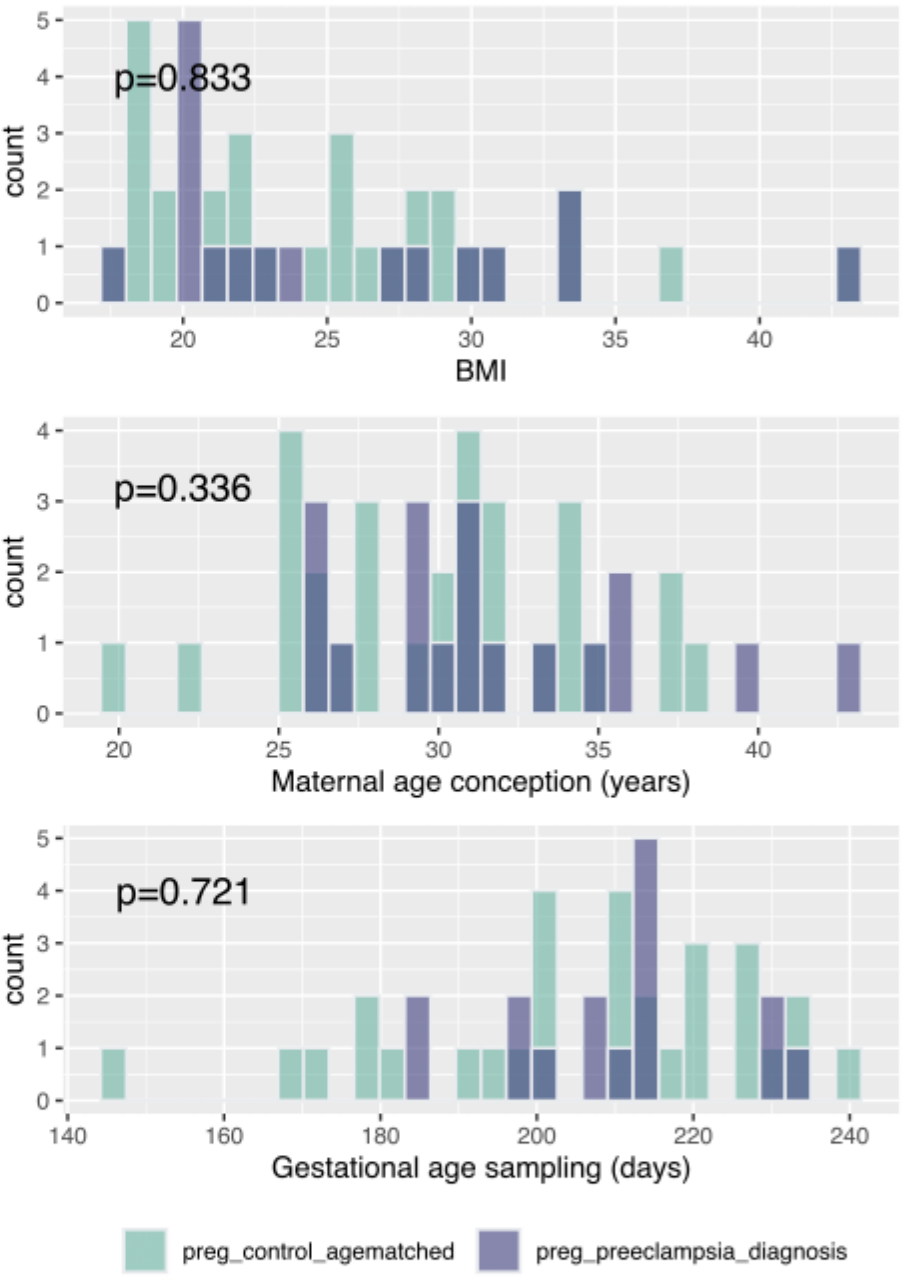

Supplement: Supplementary file 1 — Supplementary Information [file 41467_2024_46435_MOESM1_ESM.pdf]
