# Supplementary material for: Cell type signatures in cell-free DNA fragmentation profiles reveal disease biology
Source: Nat Commun. 2024 Mar 12;15:2220. doi: 10.1038/s41467-024-46435-0 (PMC10933257; doi:10.1038/s41467-024-46435-0)
Supplement: Supplementary file 3 — Reporting Summary [file 41467_2024_46435_MOESM3_ESM.pdf]

nature portfolio

Corresponding author(s): Kate E Stanley

Last updated by author(s): Aug 21, 2023

Reporting Summary

Nature Portfolio wishes to improve the reproducibility of the work that we publish. This form provides structure for consistency and transparency in reporting. For further information on Nature Portfolio policies, see our Editorial Policies and the Editorial Policy Checklist.

Statistics

For all statistical analyses, confirm that the following items are present in the figure legend, table legend, main text, or Methods section.

n/a	Confirmed	
☐	☒	The exact sample size (*n*) for each experimental group/condition, given as a discrete number and unit of measurement
☐	☒	A statement on whether measurements were taken from distinct samples or whether the same sample was measured repeatedly
☐	☒	The statistical test(s) used AND whether they are one- or two-sided<br>*Only common tests should be described solely by name; describe more complex techniques in the Methods section.*
☐	☒	A description of all covariates tested
☐	☒	A description of any assumptions or corrections, such as tests of normality and adjustment for multiple comparisons
☐	☒	A full description of the statistical parameters including central tendency (e.g. means) or other basic estimates (e.g. regression coefficient) AND variation (e.g. standard deviation) or associated estimates of uncertainty (e.g. confidence intervals)
☐	☒	For null hypothesis testing, the test statistic (e.g. *F*, *t*, *r*) with confidence intervals, effect sizes, degrees of freedom and *P* value noted<br>*Give P values as exact values whenever suitable.*
☒	☐	For Bayesian analysis, information on the choice of priors and Markov chain Monte Carlo settings
☒	☐	For hierarchical and complex designs, identification of the appropriate level for tests and full reporting of outcomes
☐	☒	Estimates of effect sizes (e.g. Cohen's *d*, Pearson's *r*), indicating how they were calculated

Our web collection on statistics for biologists contains articles on many of the points above.

Software and code

Policy information about availability of computer code

Data collection	No code or software was required to collect data
Data analysis	Code for data processing is available at https://github.com/shendurelab/cfDNA. Custom code for the case-control and predictive analyses can be accessed at https://github.com/JorisVermeeschLab/cfDNA_cell_of_origin.<br><br>The case-control and predictive analyses were performed in R (4.1.3).<br><br>Open source Rpackages:<br>data.table_1.14.8<br>ggrepel_0.9.3<br>tidyverse_1.3.2<br>Seurat_4.1.1<br>rstatix_0.7.1<br>ggplot2_3.4.2<br>e1071_1.7-12<br>pROC_1.18.0<br>viridis_0.6.2<br>Rtsne_0.15<br>igraph_1.3.0

Additional publicly available code and software used:
ichorCNA (https://github.com/broadinstitute/ichorCNA)
griffin (https://github.com/adoebley/Griffin/wiki)
shendurelab (https://github.com/shendurelab/cfDNA
bwa-mem 0.7.17-r1188
bwa-meth 0.2.2
fastp 0.12.4 and 0.20
picard 2.18.29-SNAPSHOT
samtools 1.9

For manuscripts utilizing custom algorithms or software that are central to the research but not yet described in published literature, software must be made available to editors and reviewers. We strongly encourage code deposition in a community repository (e.g. GitHub). See the Nature Portfolio guidelines for submitting code & software for further information.

Data

Policy information about availability of data

All manuscripts must include a data availability statement. This statement should provide the following information, where applicable:

- Accession codes, unique identifiers, or web links for publicly available datasets
- A description of any restrictions on data availability
- For clinical datasets or third party data, please ensure that the statement adheres to our policy

Whole-genome sequencing data generated in this study is under controlled access because patients did not consent to data deposition in public data repositories. Data is available through the European Genome-Phenome Archive under study accession number EGAS50000000178 (https://ega-archive.org/studies/EGAS50000000178) upon request to the local UZ Leuven data access committee (dac@uzleuven.be) which checks informed consent form compliance and ensures that there are no legal impediments. Requests will be handled within a month. Conflicts are handled by an independent UZ Leuven data access committee advisory board. Publicly available single cell RNA sequencing data were downloaded from Tabula Sapiens (https://tabula-sapiens-portal.ds.czbiohub.org/), Fetal Cell Atlas (https://descartes.brotmanbaty.org/bbi/human-gene-expression-during-development/), and first-trimester placenta (E-MTAB-6701). Bulk RNA sequencing data was downloaded from the Human Protein Atlas (HPA) and GTEx (https://www.proteinatlas.org/download/rna_tissue_consensus.tsv.zip). The hg38 reference genome was downloaded here http://hgdownload.soe.ucsc.edu/goldenPath/hg38/bigZips/hg38.fa.gz.

Research involving human participants, their data, or biological material

Policy information about studies with human participants or human data. See also policy information about sex, gender (identity/presentation), and sexual orientation and race, ethnicity and racism.

Reporting on sex and gender	The dataset includes 105 males and 713 females. Sex was self reported. For the 29 external colorectal cancer cases we did not have information on self reported sex.
Reporting on race, ethnicity, or other socially relevant groupings	We did not have access to race or ethnicity data from patients included in this study, except for the prospectively sampled preeclampsia cases and age-matched controls for which we have self-reported ancestry as summarized in Supplementary Table 8.
Population characteristics	The dataset includes 496 pregnant and 351 non-pregnant individuals. Of the pregnant individuals, 332 had no complications, 102 had vanishing twin syndrome, 44 had a miscarriage, and 18 had preeclampsia. Of the non-pregnant individuals, 230 were healthy, 45 had colorectal cancer, 52 had breast cancer, and 24 had multiple myeloma.<br><br>The self-reported age range of non-pregnant individuals was between 26 and 81 for breast cancer cases, between 48 and 85 for colorectal cancer cases, between 43 and 86 for multiple myeloma cases, and between 20 and 96 for healthy controls. The age range of prospectively sampled pregnant individuals was between 26 and 43 for preeclampsia cases, and between 20 and 38 for healthy controls. For the retrospective non-invasive prenatal testing samples and for the 29 external colorectal cancer cases we did not have information on age.
Recruitment	The cancer cohort is a collection of archived samples that were sampled prospectively from patients with a confirmed cancer diagnosis at UZ Leuven before initiation of treatment. Patient recruitment occurred in collaboration with the particular care and research programs of each cancer type (i.e. colorectal, breast, multiple myeloma). All patients with available data were included. The healthy non-pregnant cohort comprised individuals prospectively collected within corresponding research programs led by our group. The healthy control cohort was self-reported not pregnant and self-reported no cancer diagnosis at the time of inclusion.<br><br>The healthy pregnant cohort was randomly selected from a retrospective database of non-invasive prenatal testing (NIPT) samples that did not have known adverse pregnancy outcomes. All retrospective NIPT samples from pregnancies with a known vanishing twin (n=102) or miscarriage (n=44) with available data were included. For the prospectively sampled preeclampsia cases, healthy pregnant controls matched for maternal age, body mass index, and gestational-age were selected.<br><br>To our knowledge, there were no systematic differences, self-selection biases, or other biases in patient recruitment.
Ethics oversight	For the cfDNA samples prospectively collected, written informed consent was obtained. Non-invasive prenatal testing (NIPT) patients that were retrospectively recruited were informed about their participation through a message accompanied by an information letter in their electronic health file application. Patients were excluded from the study in case they opted out.

The study was approved by the Ethical Committee of University Hospitals Leuven, Belgium (study protocols S62285, S66450, S57999, S67127) and Vall d'Hebron Institute of Oncology (VHIO), Barcelona, Spain (study protocol PR(AG)321/2018))

Note that full information on the approval of the study protocol must also be provided in the manuscript.

Field-specific reporting

Please select the one below that is the best fit for your research. If you are not sure, read the appropriate sections before making your selection.

- [x] Life sciences
- [ ] Behavioural & social sciences
- [ ] Ecological, evolutionary & environmental sciences

For a reference copy of the document with all sections, see nature.com/documents/nr-reporting-summary-flat.pdf

Life sciences study design

All studies must disclose on these points even when the disclosure is negative.

Sample size	Sample size was determined by the availability of data (n=847)
Data exclusions	No samples were excluded from analysis.
Replication	Data were replicated in an independent validation cohort recruited from Vall d'Hebron Institute of Oncology (VHIO), Barcelona, Spain.
Randomization	Not applicable - cases were not subjected to any intervention.
Blinding	The investigators were not blinded to diagnosis in the disease cohorts because these cohorts were used for disease signature discovery and training of predictive models. We required known labels for the independent validation cohort to evaluate the performance of the trained colorectal cancer model.

Reporting for specific materials, systems and methods

We require information from authors about some types of materials, experimental systems and methods used in many studies. Here, indicate whether each material, system or method listed is relevant to your study. If you are not sure if a list item applies to your research, read the appropriate section before selecting a response.

Materials & experimental systems

n/a	Involved in the study	
[x]	[ ]	Antibodies
[x]	[ ]	Eukaryotic cell lines
[x]	[ ]	Palaeontology and archaeology
[x]	[ ]	Animals and other organisms
[ ]	[x]	Clinical data
[x]	[ ]	Dual use research of concern
[x]	[ ]	Plants

Methods

n/a	Involved in the study	
[x]	[ ]	ChIP-seq
[x]	[ ]	Flow cytometry
[x]	[ ]	MRI-based neuroimaging

Clinical data

Policy information about clinical studies

All manuscripts should comply with the ICMJEguidelines for publication of clinical research and a completedCONSORT checklist must be included with all submissions.

Clinical trial registration	*Provide the trial registration number from ClinicalTrials.gov or an equivalent agency.*
Study protocol	*Note where the full trial protocol can be accessed OR if not available, explain why.*
Data collection	*Describe the settings and locales of data collection, noting the time periods of recruitment and data collection.*
Outcomes	*Describe how you pre-defined primary and secondary outcome measures and how you assessed these measures.*
