# Supplementary material for: Cell type signatures in cell-free DNA fragmentation profiles reveal disease biology
Source: Nat Commun. 2024 Mar 12;15:2220. doi: 10.1038/s41467-024-46435-0 (PMC10933257; doi:10.1038/s41467-024-46435-0)
Supplement: Supplementary file 4 — Description of Additional Supplementary Data Files [file 41467_2024_46435_MOESM4_ESM.pdf]

Supplementary Data 1. Differentially ranked cell types in colorectal cancer patients versus matched controls. Wilcox rank sum test to compare the distribution of ranks for 456 cell types in 16 breast cancer patients versus 139 matched controls. The number of cases (n1) and controls (n2) is provided along with the test statistic and p-value. The p-value was adjusted (p.adj) using the Benjamini & Hochberg (BH) method for 456 tests. Foldchanges were calculated for each cell type by taking the ratio of the changes between the mean rank of cases and the mean rank of controls over the mean rank of cases. Cell types with an adjusted p-value < 0.05 and foldchange > 1 were annotated as differentially up-ranked. Cell types with an adjusted p-value < 0.05 and foldchange < 1 were annotated as differentially down-ranked.

Supplementary Data 2. Differentially ranked cell types in breast cancer patients versus matched controls. Wilcox rank sum test to compare the distribution of ranks for 456 cell types in 52 breast cancer patients versus 88 matched female controls. The number of cases (n1) and controls (n2) is provided along with the test statistic and p-value. The p-value was adjusted (p.adj) using the Benjamini & Hochberg (BH) method for 456 tests. Foldchanges were calculated for each cell type by taking the ratio of the changes between the mean rank of cases and the mean rank of controls over the mean rank of cases. Cell types with an adjusted p-value < 0.05 and foldchange > 1 were annotated as differentially up-ranked. Cell types with an adjusted p-value < 0.05 and foldchange < 1 were annotated as differentially down-ranked.

Supplementary Data 3. Differentially ranked cell types in multiple myeloma patients versus matched controls. Wilcox rank sum test to compare the distribution of ranks for 456 cell types in 24 multiple myeloma patients versus 90 matched controls. The number of cases (n1) and controls (n2) is provided along with the test statistic and p-value. The p-value was adjusted (p.adj) using the Benjamini & Hochberg (BH) method for 456 tests. Foldchanges were calculated for each cell type by taking the ratio of the changes between the mean rank of cases and the mean rank of controls over the mean rank of cases. Cell types with an adjusted p-value < 0.05 and foldchange > 1 were annotated as differentially up-ranked. Cell types with an adjusted p-value < 0.05 and foldchange < 1 were annotated as differentially down-ranked.

Supplementary Data 4. IchorCNA tumor fractions for cancer patients and matched controls. Estimated ichorCNA tumor fractions for patients with colorectal cancer (n=16), breast cancer (n=52), and multiple myeloma (n=24), and healthy individuals sequenced at 10-fold coverage (n=139) and <0.3-fold coverage (n=90). IchorCNA was run using the default panel of normal reference. The true group was assigned based on whether the sample was a case or control.

Supplementary Data 5. One-vs-all support vector machine for multi-class cancer classification. Decision values were generated using a support vector machine with leave-one-out cross validation and default hyperparameters. Two binary classifiers were trained: (1) breast cancer versus colorectal cancer + matched controls, and (2) colorectal cancer versus breast cancer + matched controls. Samples were assigned with a predicted class based on the classifier with the highest decision value (either breast or colorectal). Samples with decision values below the cutoff for both binary classifiers were predicted as controls. The true class refers to the sample's actual case-control status. The accuracy was calculated from the 3x3 contingency table by dividing the number of true positives and true negatives by the total number of samples. The average area under the receiver operating characteristic (ROC) curve for the two classifiers is provided.

Supplementary Data 6. Clinical information in the pregnancy cohorts. The chorionicity, study category, gestational age at sampling (weeks + days), and gestational age at fetal demise (weeks + days) is noted for pregnant individuals included in the study. For samples where the information was missing from the retrospective database "NA" is noted.

Supplementary Data 7. Clinical information in prospectively sampled preeclamptic pregnancies and matched control pregnancies. The ancestry, smoking history, chorionicity, maternal age at conception (years), height (cm), weight preconception (kg), body mass index (BMI), gestational age at sampling (weeks+days), and gestational age at sampling (days) is provided for 18 preeclamptic pregnancies that were prospectively sampled at the time of diagnosis and 30 healthy control pregnancies.

Supplementary Data 8. Differentially ranked cell types in preeclamptic pregnancies sampled at diagnosis versus matched control pregnancies. Wilcox rank sum test to compare the distribution of ranks for 496 cell types in 18 preeclampsia patients versus 30 matched controls. The number of cases (n1) and controls (n2) is provided along with the test statistic and p-value. The p-value was adjusted (p.adj) using the Benjamini & Hochberg (BH) method for 496 tests. Foldchanges were calculated for each cell type by taking the ratio of the changes between the mean rank of cases and the mean rank of controls over the mean rank of cases. Cell types with an adjusted p-value < 0.05 and foldchange > 1 were annotated as differentially up-ranked. Cell types with an adjusted p-value < 0.05 and foldchange < 1 were annotated as differentially down-ranked.

Supplementary Data 9. Differentially ranked cell types in pregnancies that had a vanishing twin versus matched control pregnancies. Wilcox rank sum test to compare the distribution of ranks for 496 cell types in 102 pregnancies that had a vanishing twin versus 301 matched control pregnancies. The number of cases (n1) and controls (n2) is provided along with the test statistic and p-value. The p-value was adjusted (p.adj) using the Benjamini & Hochberg (BH) method for 496 tests. Foldchanges were calculated for each cell type by taking the ratio of the changes between the mean rank of cases and the mean rank of controls over the mean rank of cases. Cell types with an adjusted p-value < 0.05 and foldchange > 1 were annotated as differentially up-ranked. Cell types with an adjusted p-value < 0.05 and foldchange < 1 were annotated as differentially down-ranked.

Supplementary Data 10. Differentially ranked cell types in pregnancies that went on to have a miscarriage versus matched control pregnancies. Wilcox rank sum test to compare the distribution of ranks for 496 cell types in 44 pregnancies that went on to have a miscarriage versus 301 matched control pregnancies. The number of cases (n1) and controls (n2) is provided along with the test statistic and p-value. The p-value was adjusted (p.adj) using the Benjamini & Hochberg (BH) method for 496 tests. Foldchanges were calculated for each cell type by taking the ratio of the changes between the mean rank of cases and the mean rank of controls over the mean rank of cases. Cell types with an adjusted p-value < 0.05 and foldchange > 1 were annotated as differentially up-ranked. Cell types with an adjusted p-value < 0.05 and foldchange < 1 were annotated as differentially down-ranked.
